# ﻿The rediscovery of *Caryapoilanei* (Juglandaceae) after 63 years reveals a new record from China

**DOI:** 10.3897/phytokeys.188.77242

**Published:** 2022-01-12

**Authors:** Wei-Ping Zhang, Wei-Ning Bai, Da-Yong Zhang

**Affiliations:** 1 State Key Laboratory of Earth Surface Processes and Resource Ecology and Ministry of Education Key Laboratory for Biodiversity Science and Ecological Engineering, College of Life Sciences, Beijing Normal University, Beijing 100875, China Beijing Normal University Beijing China

**Keywords:** Ailao Mountains, hickory, limestone, *Sinocarya*, Yunnan province

## Abstract

Despite having been first published in 1941, *Caryapoilanei* (A.Chev.) J.-F.Leroy is only known from three collections in Vietnam, Laos and Thailand. It has not been recollected since then and was long suspected to have become extinct through repeated deforestation events. Here, we report the rediscovery, and meanwhile the first new record in China, of this extremely rare gigantic hickory species at Yunnan province 63 years after its last collection in 1958. Three small patchy subpopulations were found with a total of about 50 adult trees having diameter at breast height (DBH) larger than 60 cm, together with some seedlings and saplings, but the fruit set was low. Based on new and fresh material, we present a revised morphological description of *C.poilanei*, and an updated distribution map for the species. In addition, we also provide a key for the hickories in China. Lastly, we suggest *C.poilanei* should be listed as a Critically Endangered (CR) species according to the latest IUCN Red List Categories and Criteria.

## ﻿Introduction

*Carya* Nutt., consisting of ca. 17 currently accepted species (Manning 1978; Chang and Lu 1979; Lu et al. 1999; Zhang et al. 2013; Grauke et al. 2016), is the second largest genus in Juglandaceae DC. ex Perleb after *Juglans* L. (Kozlowski et al. 2018), with a discontinuous distribution in South-Eastern Asia and eastern North America (Stone 1997; Lu et al. 1999). The genus *Carya* includes many internationally important and economically valuable nut crops such as pecan (*C.illinoinensis* (Wangenh.) K.Koch) and Chinese hickory (*C.cathayensis* Sarg.). All *Carya* species are monoecious with male and female inflorescences being separate, dichogamous and anemophilous, and fruit maturation process is heterochronic (Grauke and Mendoza-Herrera 2012). Based on the presence, number and arrangement of bud scales, *Carya* was divided into three sections: sect. Apocarya C.DC., sect.Carya and sect.Sinocarya Cheng & R.H.Chang (de Candolle 1864; Chang and Lu 1979). The first two sections were established in eastern North America, while the last section was found in South-Eastern Asia.

So far, five hickory species have been recognized in South-Eastern Asia, specifically in southern China, northern Vietnam, northern Laos, northern Thailand and north-eastern India (Manning 1963; Chang and Lu 1979; Srisanga 2017). Among the five species, three (*C.cathayensis*, *C.hunanensis* W.C.Cheng & R.H.Chang and *C.kweichowensis* Kuang & A.M.Lu) are endemic to China; their distributions hardly overlap and are, in general, extremely rare (Chang and Lu 1979; Lu et al. 1999; Grauke and Mendoza-Herrera 2012). *Caryatonkinensis* Lecomte seems to be the most widespread species in South-Eastern Asia, distributed in southwest China, northern Vietnam, northern Thailand and north-eastern India (Manning 1963; Chang and Lu 1979; Srisanga 2017). *Caryapoilanei* (A.Chev.) J.-F.Leroy was described in 1941 based on a single collection from northern Vietnam in 1937 (Chevalier 1941; Leroy 1950), and later, Manning (1963) added a record of specimen collected from Laos in 1932. Surprisingly, through the GBIF (Global Biodiversity Information Facility) network, we came across a specimen collected from Thailand in 1958 (*Smitinand 4319*, L0069301/L.1551797), which was identified as *C.poilanei* by Michael Vomberg in 2006 and has not yet been recorded in Flora of Thailand. Although botanists have made efforts to seek the surviving members in the area where the type tree was originally located (Grauke et al. 1991; Grauke et al. 2016), living trees of *C.poilanei* have not been found for over 63 years, and thus this species has been suspected to be extinct in the wild (Grauke and Mendoza-Herrera 2012; Grauke et al. 2016).

**Figure 1.**
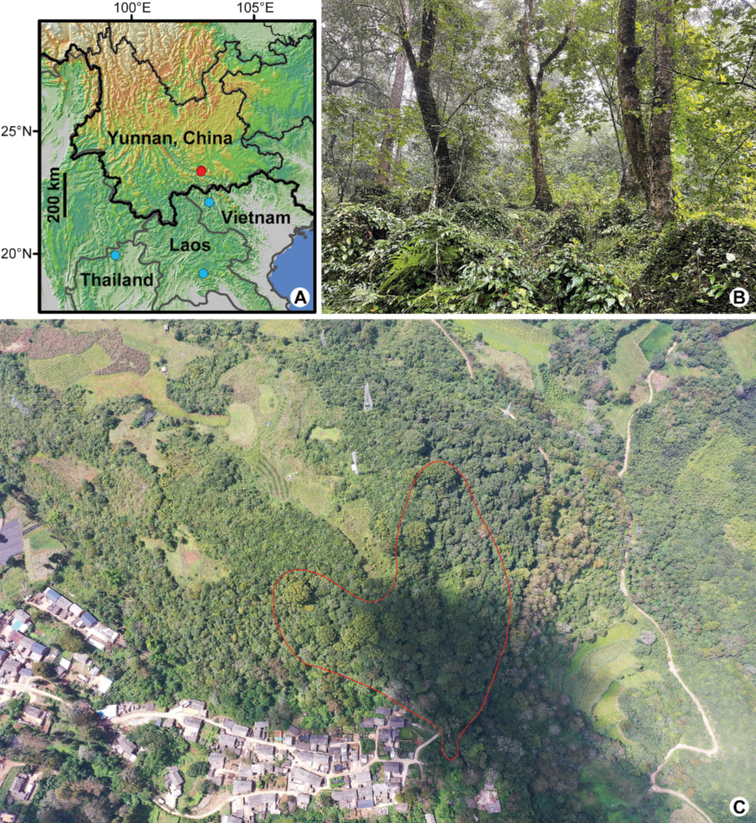
Distribution and habitat of *Caryapoilanei* (A.Chev.) J.-F.Leroy **A** four distribution sites in Yunnan province of China, Vietnam, Laos and Thailand. Red circle indicates the localities taken from the new record areas, and blue circles indicate historical distribution localities where the trees have been presumably extirpated **B** the limestone mountain habitat **C** eastern edge of Ailao Mountains, with human habitation in plateau. Red lines mark the boundary of the *C.poilanei* distribution in Dajinglaozhai Village.

**Figure 2.**
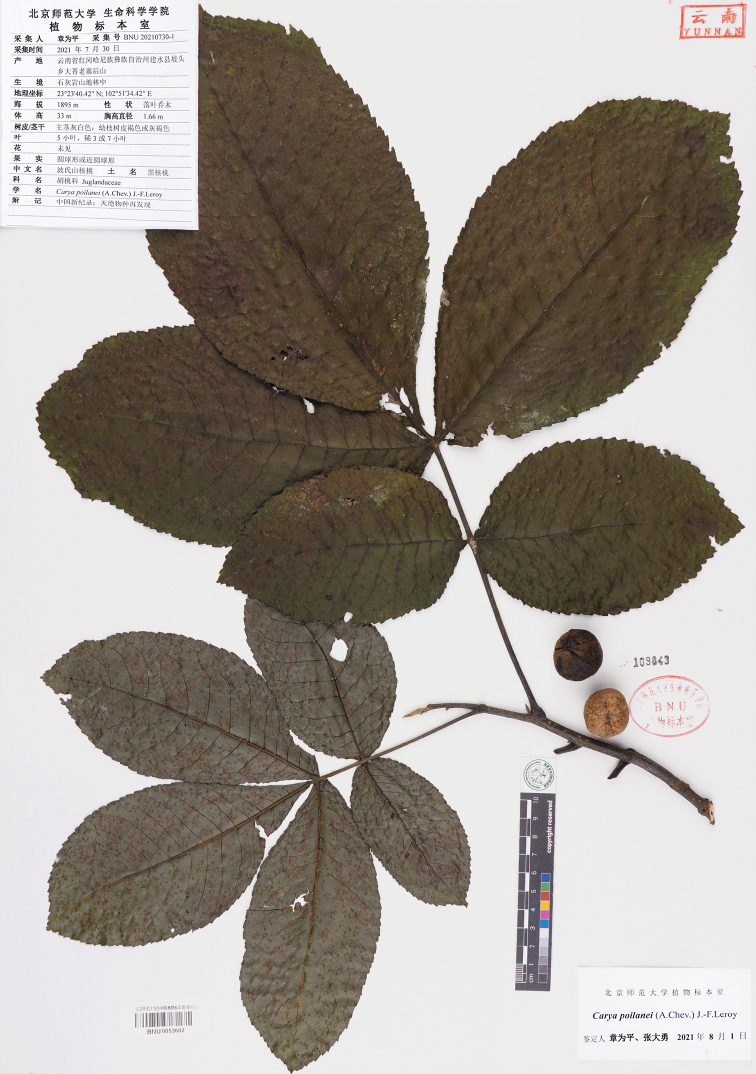
*Caryapoilanei* (A.Chev.) J.-F.Leroy (representative specimen, BNU20210730-1, BNU).

**Figure 3.**
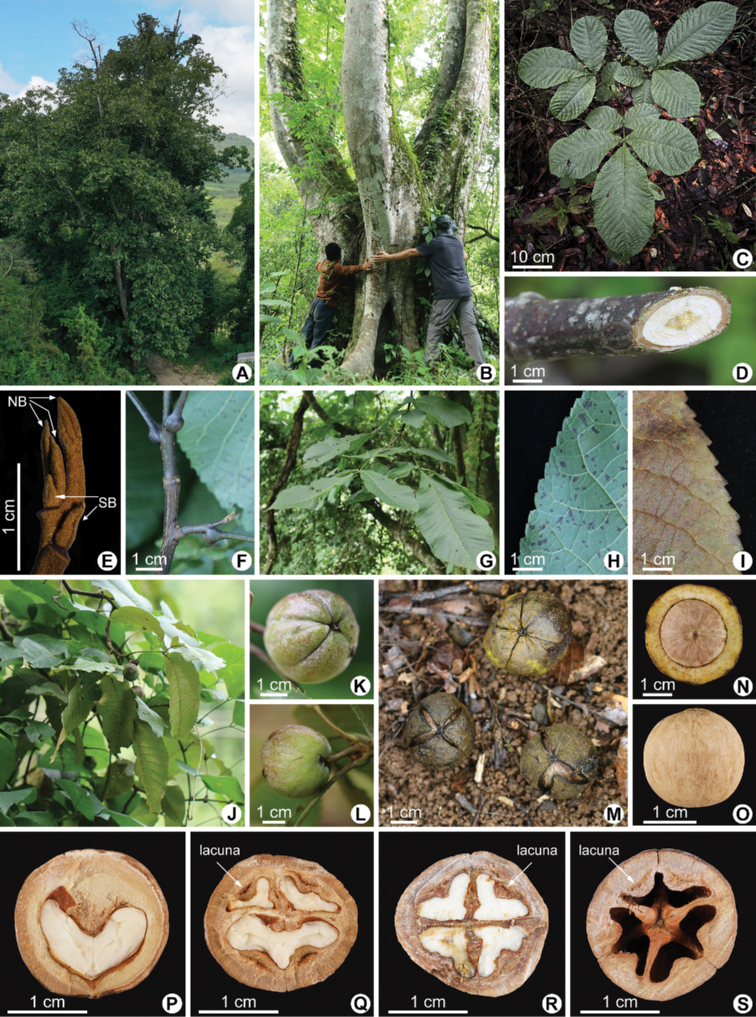
Living plants of *Caryapoilanei* (A.Chev.) J.-F.Leroy **A** tree **B** trunk, with a maximum diameter at breast height (DBH) of 1.97 m **C** sapling **D** twig **E** terminal buds (NB: naked buds; SB: buds with valvate scales) **F** petiole enlarged at base **G** leaf, showing 5 leaflets **H** leaf abaxial glabrous **I** leaf abaxial densely brown scales **J** fruiting branch **K** husk, showing base **L** husk, showing peduncle **M** husk, irregularly dehiscent **N** shell, vertical view **O** shell, lateral view **P** longitudinal section of nut **Q–S** transversal section of nuts, with 3, 4 and 6 chambers, respectively; lacunae present.

At the end of July 2021, during a scientific field trip in Jianshui County, southern Yunnan province of China, three fragmented subpopulations of *Carya* were discovered near the eastern edge of Ailao Mountains (Fig. 1). After morphological comparison to the images of type material and scrutiny of the brief description, we confirm that they belong to *C.poilanei* (Figs 2, 3). This finding allowed us to update its morphological description, discuss its geographic distribution, and assess its conservation status. Furthermore, it would be conducive to inferring its phylogenetic position within *Carya*, and valuable to exploit its genetic resources for breeding and crop development in future days.

## ﻿Materials and methods

Specimens were collected in the field of Jianshui county, Yunnan province in July to October, 2021. Except for Fig. 1C and Fig. 3A which were taken by DJI Mavic 2 Pro, the rest of the photos were taken by Canon EOS 70D with Sigma 17–50 mm (f/2.8 EX DC OS HSM) and Canon EF 100 mm (f/2.8L IS USM) lens. Because these four months are the fruiting period, we were unable to investigate the flower phenology and characters. The morphology of the species was observed and measured based on living plants and dry specimens. Morphological measurements for more than six freshly differentiated samples from the adult trees were taken using both a ruler and a digital caliper. All herbarium voucher specimens collected by us are deposited in the Herbarium of College of Life Science, Beijing Normal University (BNU).

## ﻿Taxonomy

### ﻿ Carya poilanei

Taxon classificationPlantaeFagalesJuglandaceae

(A.Chev.) J.-F.Leroy, Rev. Int. Bot. Appl. Agric. Trop. 30: 428. 1950.

51D4A769-0D4F-52C4-8B04-2D3AB7255C93

Figures 2 , 3

Juglans poilanei A.Chev., Rev. Bot. Appl. Agric. Trop. 21: 496. 1941.

#### Type.

Vietnam. Lai-Chau province, within the great forest near the slopes of Pou-Nhou, in calcareous soil, at 1000 m. elev., 31 Dec. 1937, *Poilane 26964*, (Holotype: P [barcode P00605884, image!]; isotypes: P [barcode P00223582, P00605885, P00605886, image!]).

#### Revised description.

Tree up to 15–40 m tall, deciduous, monoecious. Trunk to 0.5–2 m in diam.; bark smooth or somewhat rough, gray to whitish. Branches brown or gray-brown, initially with orange-yellow glandular and white pilose above, later almost glabrous and sparsely glandular, with roughish, scattered lenticels; pith solid in stem. Terminal buds 3–15 mm, both naked and with valvate scales, but the scales often drop easily, gray brown or brown. Leaf length 30–60 cm (incl. petiole), imparipinnate, soft green, papery; petiole 6–12 cm, enlarged at base, pubescent or glabrous; rachis pubescent or glabrous, sparsely glandular; leaflets (3 or) 5 (or 7), apical one shortly petiolulate, terminal petiolule 5–12 mm, lateral ones sessile or subsessile, broad obovate, occasionally obovate lanceolate or ovate-lanceolate, base skewed to nearly round, apex shortly obtuse or acuminate, margin serrate; adaxially smooth or finely scabrid, abaxially glabrous except for hairs along midvein and in axils of secondary veins, secondary vein 15–25 pairs, sometimes old leaflets blade densely covered with brown scales; apical and middle leaflets 25–40 × 12–20 cm, much larger than base leaflets. Flowers not seen. Fruits subglobose or compressed-globose, 2.8–3.2 × 3–3.5 cm, with peduncle, 1.5–6 cm length; husk wingless, sparely orange-glandular, shortly pubescent, 3.6–5.6 mm thick, moderate keels extending to middle, cracks into 3 or 4 petals when dried; shell subglobose, closely white tomentose, with 2 longitudinal ridges, apex slightly convex, 2.8–3.5 mm thick, cracks into 2–4 sections when dried, equal or unequal; 3, 4 or 6 chambered at base, lacunae present in the wall near the secondary septa. Flower unclear. Fruit Sep. Germination hypogeal.

#### Distribution.

China: Yunnan Province, Jianshui County; Vietnam: Lai-Chau Province, Pou-Nhou; Laos: Vientiane Province, Ban Mouang Cha (Muang Cha); Thailand: Chiang Mai (Chiengmai) Province, Fang District, Doi Pha Hom Pok Range (Fig. 1A).

#### Habitat and ecology.

It grows on southeastern and southern slope of the limestone or calcareous mountain at an elevation of 1000–2050 m (Fig. 1B, C). The three sites we discovered are in the subtropical region, on the eastern edge of Ailao Mountain, and 15 kilometers to the south are hot dry valleys (elevation about 200 m). Of these three distribution points, the closest distance between the two points is about 2 km, while the farthest is just about 6 km. Among the three threatened relic forests, *C.poilanei* are dominant and impressive trees.

#### Additional specimens examined.

**China**: Yunnan Province, Jianshui County, Potou Town, Dajinglaozhai Village, on limestone, 23°23'40.42"N, 102°51'34.42"E, 1895 m, 30 Jul. 2021, *Zhang BNU20210730-1* (BNU, [barcode BNU0053602]) (Fig. 2), ibid., 23°23'42.33"N, 102°51'36.65"E, 1889 m, 31 Jul. 2021, *Zhang 20210731-30* (BNU, KUN); Ximatang Village, on limestone, 23°24'5.68"N, 102°52'17.38"E, 2019 m, 31 Jul. 2021, *Zhang 20210731-3* (BNU), ibid., 23°24'5.28"N, 102°52'11.25"E, 1980 m, 31 Jul. 2021, *Zhang 20210731-14* (BNU); Yuchu Village, on limestone, 23°20'47.75"N, 102°51'35.90"E, 1892 m, 31 Jul. 2021, *Zhang 20210731-17* (BNU, KUN), ibid., 23°21'44.46"N, 102°51'27.65"E, 1859 m, 5 Oct. 2021, *Zhang 20211005-1* (BNU). **Laos**: Vientiane Province, Ban Mouang Cha (Muang Cha), on rocky limestone hill, c. 1500 m, 16 Apr. 1932, *Kerr 21092* (BM [barcode BM013822350, image!], K, P [barcode P06811763, image!]). **Thailand**: Chiang Mai (Chiengmai) Province, Fang District, Doi Pha Hom Pok Range, 19°55'0.80"N, 99°12'52.20"E, 1100–1180 m, 23 Feb. 1958, *Smitinand 4319* (L [barcode L0069301/L.1551797, image!]).

### ﻿Key to the five native species of *Carya* Nutt. in China

**Table:** 

1	Grows on limestone mountain; leaflets (3) 5 (7); nuts compressed-globose; husk smooth without longitudinal ridges, sparely orange-glandular; lacunae present in nutshell	**2**
–	Grows on mountain slopes, valleys and riverbanks; leaflets 5–7 (9); nuts obovoid, ellipsoid or subglobose; husk wrinkled with longitudinal ridges; densely orange-glandular; lacunae absent in nutshell	**3**
2	Leaflets mostly broad obovate, papery, rough, margin serrate	***C.poilanei***
–	Leaflets elliptic to elliptic-lanceolate, more or less leathery, glossy, margin obtusely serrate	***C.kweichowensis***
3	Petiole tomentose; husk slightly winged	***C.tonkinensis***
–	Petiole glabrescent; husk conspicuously winged	**4**
4	Husk winged to middle	***C.hunanensis***
–	Husk winged to base	***C.cathayensis***

## ﻿Discussion

Initially, French botanist Auguste Chevalier placed *C.poilanei* into the genus *Juglans* after he observed the only specimen collected from Vietnam in 1941 (Chevalier 1941). The possible reason is that *C.poilanei* has the lacunae character (Fig. 3Q-R) which superficially resembles some species in the genus *Juglans*. Subsequently, Leroy (1950) placed the species to the genus *Carya*, based on the morphological features easy to distinguish from the *Juglans* genus such as unicular strands in the shell (Fig. 3O) and basal plexus, solid rather than chambered pith in the stem (Fig. 3D) (Leroy 1955). Although the presence of lacunae in the septum and/or shell walls are an atypical characteristic of relict hickory species, it has been recorded in seven fossil species from Europe (Mai 1981), which may help to better understand the biogeographic histories of *Carya*. Besides, we also see evident lacunae in the shell walls of *C.kweichowensis*, which seems to be related to *C.poilanei* as it is also located in limestone mountain habitat (Fig. 1B, C). However, *C.poilanei* differs from *C.kweichowensis* in having broad obovate leaflets (Figs 2, 3C, G, J vs. elliptic to elliptic-lanceolate leaflets in *C.kweichowensis*) and gray brown or brown buds (Fig. 3E vs. black buds in *C.kweichowensis*) (Chang and Lu 1979). Significantly, the terminal buds of *C.poilanei* are not uniform, both naked and protected by valvate scales (Fig. 3E), but the scales are relatively small and easy to drop off. Molecular data would be necessary to explore its systematic status in the future work.

The hickory trees are not found in any nature reserve, but in the back hills of some aged village (Fig. 1C). Fortunately, these trees are close to villages and tall enough to be regarded as sacred trees by local villagers, and hence saved from being deforested. Based on our fieldwork in these areas, we found a total of three small and fragmented subpopulations, preserving about 50 adult trees with diameter at breast height (DBH) larger than 60 cm (Fig. 3B) as well as some understory seedlings (Fig. 3C) and juvenile trees; however, fruit sets were low. We evaluated the conservation status for the *C.poilanei* according to the latest IUCN Red List guidelines (IUCN Standards and Petitions Committee 2019) and suggested that the species should be ranked as critically endangered (CR). Meanwhile, we recommend that the species should be added to the new version of the List of National Key Protected Wild Plants and Plant Species with Extremely Small Populations, China. Given its rather limited number of individuals and narrow potential geographical range, this species clearly needs to be properly protected, even in the absence of known strategies of utilization. More efforts are required for strengthening its *in situ* and *ex situ* conservation, as well as studying its systematic position and genetic diversity. We propose that, in the future, protected areas should be established *in situ*, and a more exhaustive investigation could be launched into the nearby limestone mountains.

## Supplementary Material

XML Treatment for Carya poilanei

## Supplementary materials

Supplementary material 1 The rediscovery of *Caryapoilanei* (Juglandaceae) after 63 years, a extremely gigantic hickory species new record from China Data type COL Explanation note Despite having been first published in 1941, *Caryapoilanei* (A.Chev.) J.-F.Leroy is only known from three collections in Vietnam, Laos and Thailand. It has not been recollected since then and was long suspected to have become extinct through repeated deforestation events. Here, we report the rediscovery, and meanwhile the first new record in China, of this extremely rare gigantic hickory species at Yunnan province 63 years after its last collection in 1958. Three small patchy subpopulations were found with a total of about 50 adult trees having diameter at breast height (DBH) larger than 60 cm, together with some seedlings and saplings, but the fruit set was low. Based on new and fresh material, we present a revised morphological description of *C.poilanei*, and an updated distribution map for the species. In addition, we also provide a key for the hickories in China. Lastly, we suggest *C.poilanei* should be listed as a Critically Endangered (CR) species according to the latest IUCN Red List Categories and Criteria. https://binary.pensoft.net/file/633251This dataset is made available under the Open Database License (http://opendatacommons.org/licenses/odbl/1.0/). The Open Database License (ODbL) is a license agreement intended to allow users to freely share, modify, and use this Dataset while maintaining this same freedom for others, provided that the original source and author(s) are credited.Wei-Ping Zhang
