# Supplementary material for: ﻿The rediscovery of Caryapoilanei (Juglandaceae) after 63 years reveals a new record from China
Source: PhytoKeys. 2022 Jan 12;188:73–82. doi: 10.3897/phytokeys.188.77242 (PMC8770418; doi:10.3897/phytokeys.188.77242)

Dear committee of Catalogue of Life:

I enclose a manuscript entitled ‘The rediscovery of *Carya poilanei* (Juglandaceae) after 80 years reveals a new record from China’ for possible publication in Phytokeys.

Some long-disappeared species will really go extinct before they are rediscovered. Despite having been first published in 1941, *Carya poilanei* (Juglandaceae) is only known from two original collections in Vietnam and Laos; however, it has not been recollected since then and long suspected to be extinct by repeated deforestation events. Here, we report the rediscovery, and meanwhile the first new record in China, of this extremely rare species at Yunnan province after 80 years. Our rediscovery allowed us to update its morphological description, discuss its geographic distribution, and assess its conservation status. Furthermore, it would be conducive to inferring its phylogenetic position within *Carya*, and valuable to exploit its genetic resources for breeding and crop development in future days.

We feel that our work has broad appeal because it reintroduces a gigantic hickory tree growing up to 40 m tall with diameter at breast height max to 1.97 m in nature.

Yours sincerely,

Wei-Ping Zhang

(on behalf of all coauthors)


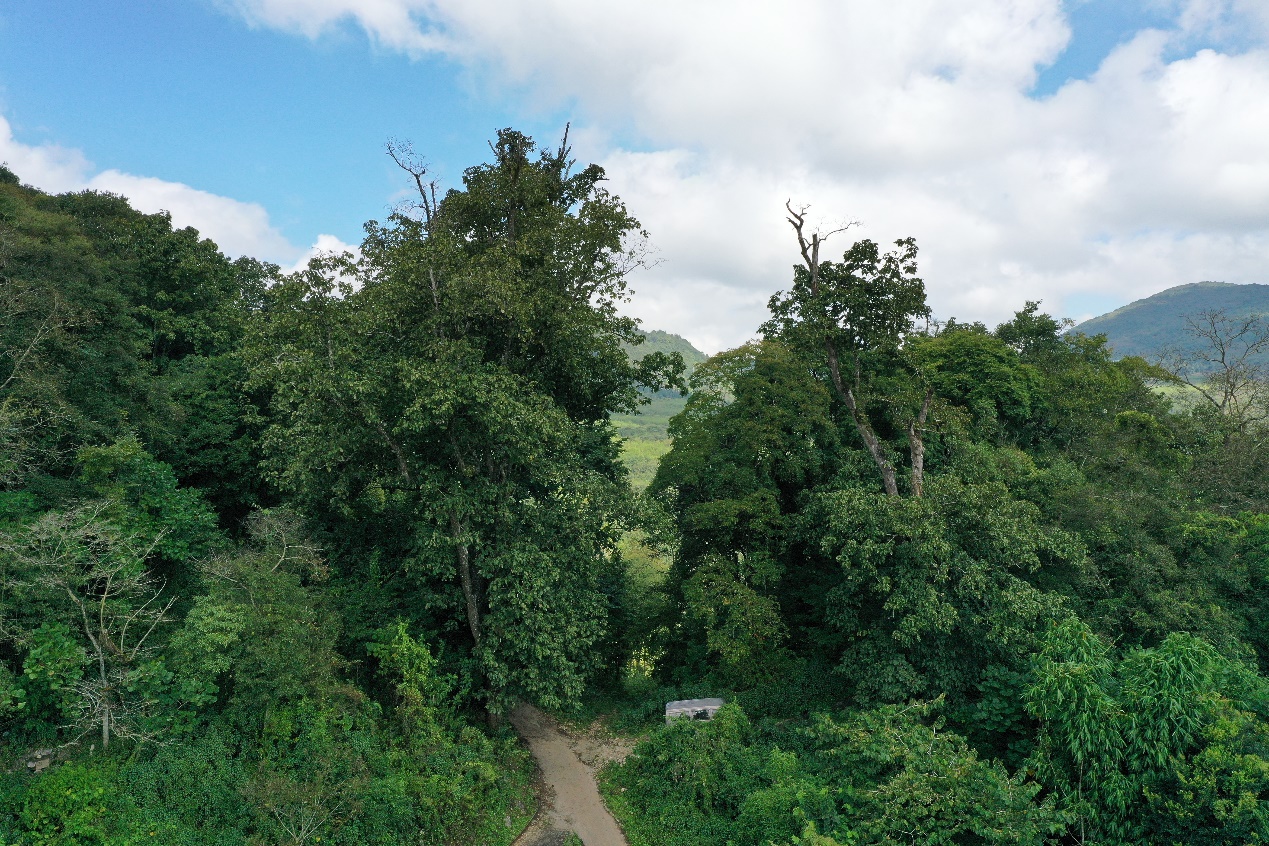


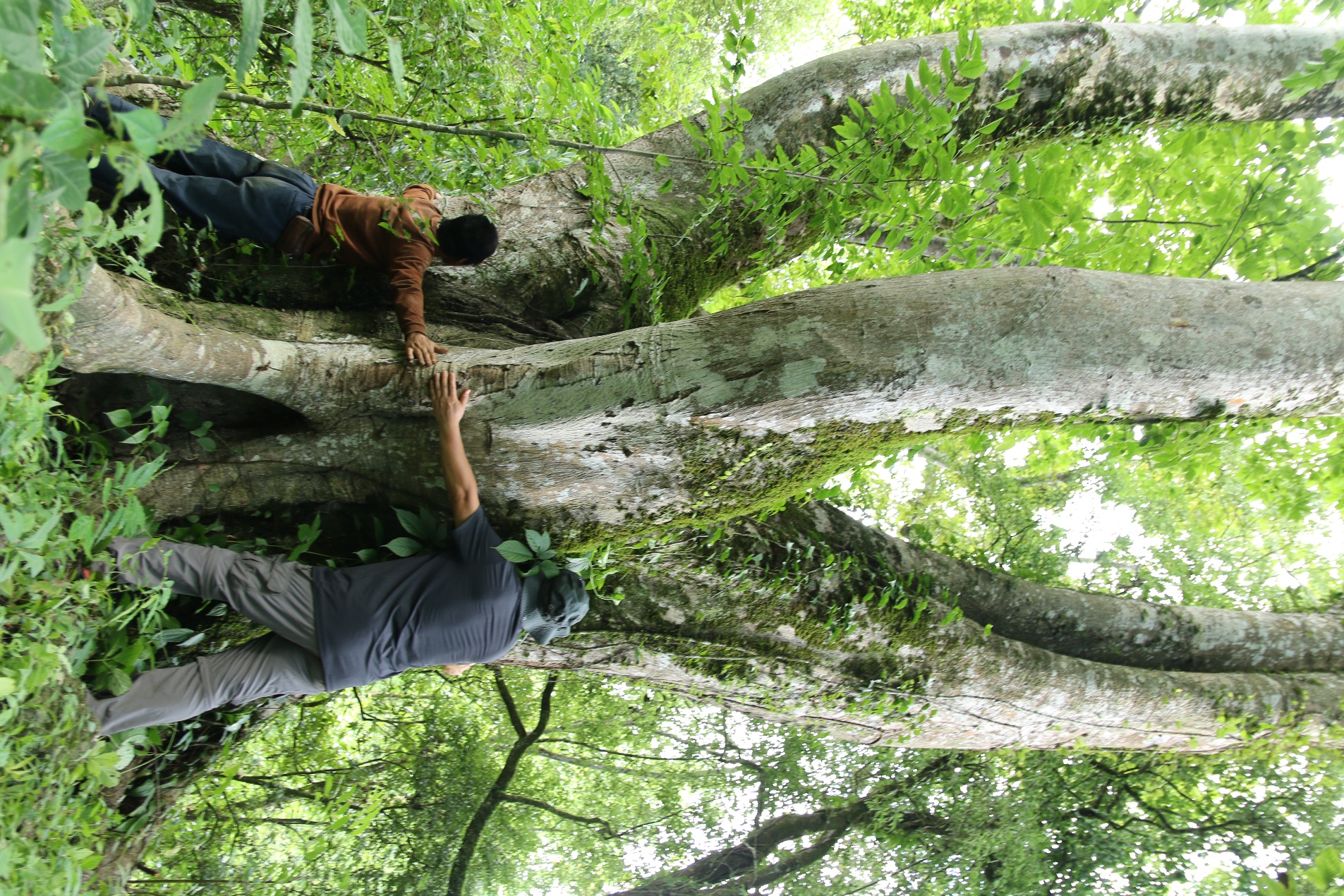

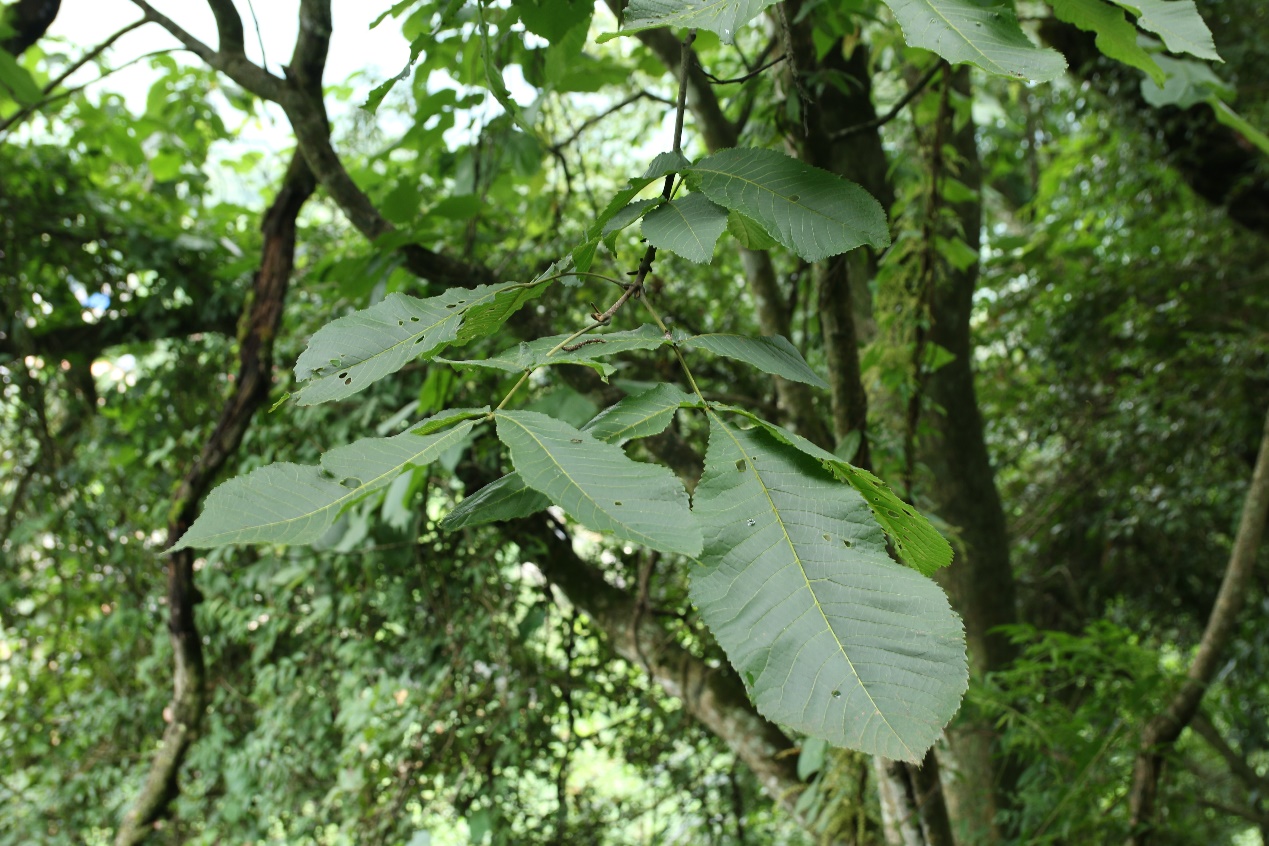


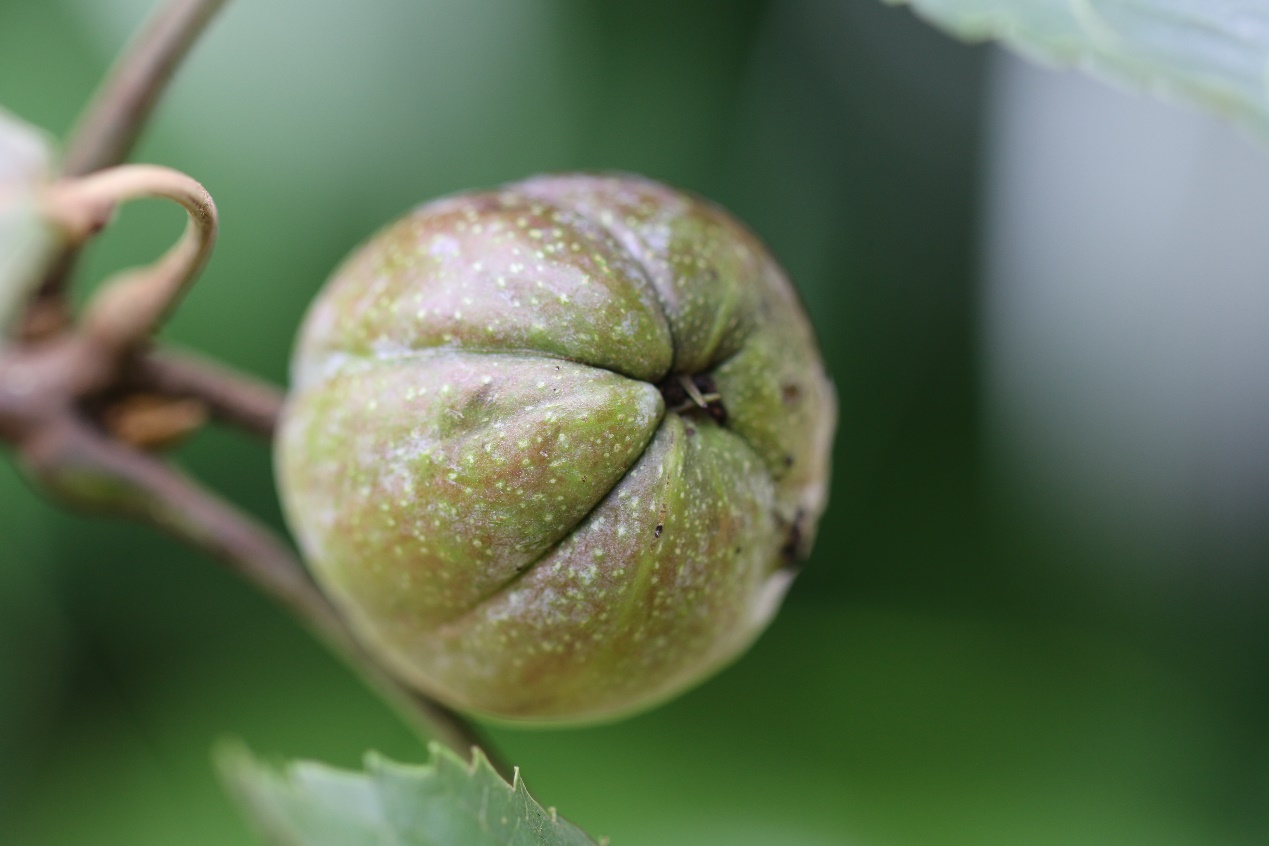

Supplement: Supplementary material 1 — The rediscovery of Caryapoilanei (Juglandaceae) after 63 years, a extremely gigantic hickory species new record from China [file phytokeys-188-073-s001.docx]
